# Implications of COVID-19 vaccine boosters amid the emergence of novel variants of SARS-CoV-2

**DOI:** 10.1016/j.amsu.2022.103612

**Published:** 2022-04-12

**Authors:** Manish Dhawan, Talha Bin Emran, Om Prakash Choudhary

**Affiliations:** Department of Microbiology, Punjab Agricultural University, Ludhiana, 141004, India; Trafford College, Altrincham, Manchester, WA14 5PQ, UK; Department of Pharmacy, BGC Trust University Bangladesh, Chittagong, 4381, Bangladesh; Department of Veterinary Anatomy and Histology, College of Veterinary Sciences and Animal Husbandry, Central Agricultural University (I), Selesih, Aizawl, 796015, Mizoram, India

**Keywords:** Boosters, COVID-19, SARS-CoV-2, Variants, Vaccines

Dear Editor,

The COVID-19 pandemic has wreaked havoc throughout the world. Furthermore, the scientific community is concerned about the evolution of its causative agent, i.e., SARS-CoV-2, and the emergence of novel SARS-CoV-2 variants [1]. Many vaccines and therapeutic regimens have been developed quickly to address the negative repercussions of the COVID-19 pandemic. Vaccines have played a significant role in establishing adequate humoral and cell-mediated immune responses among a wide range of populations [1,2]. Moreover, upon receiving the complete primary vaccination with the currently available COVID-19 vaccines, booster doses of vaccine may be required for a variety of reasons, including the dwindling of the protective immune response, the advent of novel strains of SARS-CoV-2 that evade vaccine-induced protection, and insufficient immune responses in specific risk communities, such as immunocompromised individuals [3]. In this context, booster doses have been demonstrated to be effective in boosting the levels of SARS-CoV-2-specific neutralizing antibodies (nAbs) that are cross-reactive to contemporary VOCs (Variants of Concern) and VOIs (Variants of Interest). It is important to note that the administration of the booster doses is crucial for elderly and immunocompromised people at the highest risk of severe COVID-19 infection [4].

Many COVID-19 vaccinations have been employed, including mRNA, viral vector, inactivated, and protein-based vaccines, to provide boosters [5]. Interestingly, a booster dose against SARS-CoV-2 can significantly enhance nAbs levels or alternative indicators such as anti-spike IgG. For the population unable to react to the conventional vaccination, such as immunocompromised people, a booster dosage can help induce optimum concentration of nAbs against SARS-CoV-2 [4,5]. A booster enhances the number of antibodies (Abs) with cross-reactivity towards novel variants of SARS-CoV-2, and the optimum concentration of the Abs at the time of infection can provide significant level protection. This data implies that a high concentration of nAbs maintained by repeated vaccinations is enough to prevent COVID-19 produced by emerging variants of SARS-CoV-2 such as Delta and Omicron variants [4]. According to recent real-world evidence from Israel, the third dosage of an mRNA vaccine significantly lowered the chance of infection or serious illness caused by the Delta variant [5]. Hence, the provision of many countries providing booster doses may substantially influence the worldwide pandemic's course [4].

On the other hand, current vaccinations provide very effective and long-lasting protection against COVID-19-related hospitalizations and fatalities in people of all ages, indicating that a booster dose may not be required for all fully vaccinated people. Furthermore, achieving herd immunity necessitates fair vaccination distribution and access worldwide. Booster dose administration costs may prolong inequitable global vaccination distribution and exacerbate vaccine apprehension [4]. Rather than squandering time and money on large-scale boosters, which are less likely to benefit global health than primary vaccination in an under-vaccinated region, the current focus should be on protecting vulnerable populations as soon as feasible [3,4].

Another difficulty has been identified as discrepancies in vaccination distribution among nations, which has led to the emergence of SARS-CoV-2 variants. There are still many unprotected, vulnerable groups worldwide that need to be vaccinated as soon as possible. Other VOCs may evolve in the future if SARS-COV-2 continues to circulate, particularly among the unvaccinated, and the resultant viral evolution may eventually lead to vaccine-resistant variants [6]. As a result, public health professionals and policymakers should address these aspects when selecting which populations must get boosters and when they should be given [[4], [5], [6]].

## Ethical approval

This article does not require any human/animal subjects to acquire such approval.

## Sources of funding

This study received no specific grant from any funding agency in public, commercial, or not-for-profit sectors.

## Author contribution

**Manish Dhawan:** Conceptualization, Data Curation, Visualization, Writing - Original Draft, Writing - review & editing. **Talha Bin Emran:** Data Curation, Writing - Original Draft, Writing - review & editing **Om Prakash Choudhary:** Visualization, Writing - Original Draft, Writing - review & editing.

## Consent

Not Applicable.

## Trial registry number

1. Name of the registry: Not applicable
2. Unique Identifying number or registration ID: Not applicable
3. Hyperlink to your specific registration (must be publicly accessible and will be checked): Not applicable

## Guarantor

Mr. Manish Dhawan, ^1^Department of Microbiology, Punjab Agricultural University, Ludhiana 141004, India; ^2^Trafford College, Altrincham, Manchester WA14 5PQ, UK; Email Address: dhawanmanish501@gmail.com; Contact No: 01619287242.

## Declaration of competing interest

All authors report no conflicts of interest relevant to this article.

## References

[1] Dhawan M., Priyanka O.P. Choudhary (2022). Omicron SARS-CoV-2 variant: reasons of emergence and lessons learnt. Int. J. Surg..

[2] Dhama K., Dhawan M., Tiwari R., Emran T.B., Mitra S., Rabaan A.A., Alhumaid S., Alawi Z.A., Al Mutair A. (2022). COVID-19 intranasal vaccines: current progress, advantages, prospects, and challenges. Hum. Vaccines Immunother..

[3] Priyanka O.P., Choudhary I. Singh (2022). Adjudicating the logistics of COVID-19 vaccine boosters from a global perspective. Hum. Vaccines Immunother..

[4] Burckhardt R.M., Dennehy J.J., Poon L.L.M., Saif L.J., Enquist L.W. (2022). Are COVID-19 vaccine boosters needed? The science behind boosters. J. Virol..

[5] Bar-On Y.M., Goldberg Y., Mandel M., Bodenheimer O., Freedman L., Kalkstein N., Mizrahi B., Alroy-Preis S., Ash N., Milo R., Huppert A. (2021). Protection of BNT162b2 vaccine booster against covid-19 in Israel. N. Engl. J. Med..

[6] Dhawan M., Priyanka A. Sahni, Choudhary O.P. (2022). Vaccine inequity and hesitancy: dual factors in the emergence of novel SARS-CoV-2 variants. Ann. Med. Surg..

